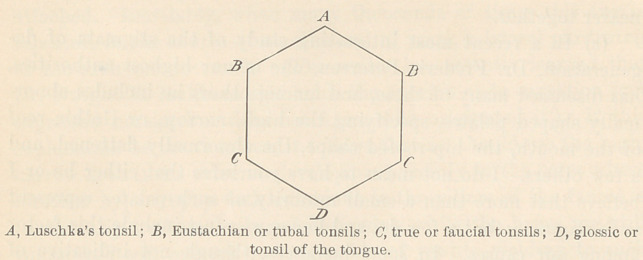# False Tonsils

**Published:** 1899-06

**Authors:** Robert H. M. Dawbarn

**Affiliations:** New York


### International Dental Journal.

Vol. XX. June, 1899. No. 6.

### Original Communications.[1]

### FALSE TONSILS.[2]

BY ROBERT H. M. DAWBARN, M.D., NEW YORK.[3]

The subject upon which I have been asked to address you this evening is in one respect unique.

Nowhere else, I verily believe, than within the magic circle of "Waldeyer's tonsillar ring"[4] can be found a common meeting-ground for seven distinct classes of practitioners, all really interested in the subject.

The otologist, because here lies the commonest cause of deafness. The rhinologist, because here is found one of the most frequent sources of nasal catarrh. The laryngologist, for the reason, among others, that obstruction of the vault interferes wofully with tone-production, as also do the high narrow arch of the palate and abnormally small antra. The stomatologist, because the disease in question leads to peculiar malformations of the upper jaw and to

[1] The editor and publishers are not responsible for the views of authors of papers published in this department, nor for any claim to novelty, or otherwise, that may be made by them. No papers will be received for this department that have appeared in any other journal published in the country.

[2] Read at a meeting of The New York Institute of Stomatology, held March 7, 1899.

[3] Professor of Surgery and of Surgical Anatomy, New York Polyclinic Medical School and Hospital; Surgeon to the New York City Hospital.

[4] "Waldeyer: Ueber den lymphatischen Pharynxring." Deutsche Medicinische Wochenschrift, No. 20, 1884.

defective dentition, with consequent necessity for regulating-work. The neurologist, because a half-dozen psychoses and neuroses have an occasional starting-point here, from reflex irritation, as well as from a poisoned and ill-aërated blood which cannot properly nourish the nervous centres. The general practitioner, because of effects upon all organs and bodily functions from the cause just hinted at; and, finally, the general surgeon diffidently expresses his interest in the subject—just because.

I believe that all surgeons commonly operate for removal of tonsils of all abnormal types and in all situations. Nevertheless, the field for general surgery is narrowing as rapidly as that for general medicine to-day; and we can hardly tread without stepping upon some brother's special field. Hence the diffidence aforesaid.

As evidence in one specialty alone of this general tendency to elbow us off the face of the earth, a well-known surgeon remarked to the writer not long since that gynæcology is steadily advancing uphill; he is keeping his weather eye upon it.

Time was when it was limited above by the brim of the true pelvis; now it is nearer the brim of the hat. They are rising in the world; and at present nothing short of a line firmly drawn just beneath the chin seems likely to stop their upward march. Maybe they too will find excuse for entering the magic circle of Waldeyer!

I have spoken of pharyngeal false tonsils. The correct term is pharyngeal lymphoids, or lymphoid growths. Adenoids is the more usual designation, but there is no pathological justification for it, and writers careful in nomenclature are already dropping this unfortunate term.

These vegetations are practically identical in structure with the faucial tonsils. That is the same as saying that they are open-meshed lymph-nodes, very vascular, having, however, no deep crypts or pockets, but abundance of irregular spaces between them wherein dirt and discharges are held, and being in a measure without the dense capsular investment of lymph-nodes elsewhere.

At what age do they appear, and with what frequency? There seems to be no period even of early infancy exempt. One of the worst cases I have cured was in a baby not nine months old. A surprisingly large amount of lymphoid material was removed.

Regarding the frequency, doubtless climate is a prominent factor therein. When, less than a generation ago, the disease began to be generally recognized, it was at first claimed that five per cent.

of all children suffer therefrom, to some degree, in our very severe climate. But of late I am sure that this estimate is by those competent to judge regarded as much too low. Some specialists double it; and yet I can well believe that in the tropical and sub-tropical zones, and especially where the air is comparatively dust-free and the variations in temperature and humidity are less startling than with us, there may be much lower percentages than I have named.

The ill effects of large pharyngeal and faucial tonsils are numerous. With an audience of stomatologists I will, however, only discuss and dwell upon those having a direct bearing upon that specialty of medicine.

Perhaps the most prominent of these bad results are four:

1. The high narrow arch of the palate.
2. The insufficient development of the upper jaw.
3. The dentition, irregular in order and imperfect in quality.
4. The tendency to decay, especially of the teeth nearest these growths, and to various types of stomatitis and gingivitis, mainly induced by the myriad microbes which the interspaces of these vegetations harbor and encourage.

1 and 2. To study these in order: How shall we explain the high, narrow hard palate which is so frequently found in children subject to the troubles in question? After considerable thought I would reply, that there seem to be at least six factors in its causation.

(*a*) The first and most important of these I believe to be a purely mechanical one. To explain: I am surely not overstating the fact if I say that nine out of every ten cases of pharyngeal tonsils are also cases of diseased and abnormally large true or faucial tonsils.

It is certainly the rule with me rather than the exception to have to remove these at the same operation with the emptying of the obstructed pharynx.

Now, I am certain that any other surgeon will agree in telling you that a fact too frequently overlooked is the firm adhesion so often found, when sought for, between the diseased tonsil and the pillars of the fauces, and especially the anterior pillar. An operator neglects his duty who does not look for and carefully separate such adhesions; and sometimes this is not easily done, for they twain have become one flesh, so to speak.

This being true, it seems plain that in the act of swallowing, the large tonsils, being dragged down mechanically with the bolus of solid food in the act of swallowing, in turn exert a distinct downward pull or tug upon their adherent pillars; and these in turn must pull down upon the sides of the bony palate, to which they are attached. Inevitably, when many thousands of times this tug is repeated, in the course of months and of years, a lateral narrowing of the arch has to result. (The writer has recently learned that Dr. Dwight L. Hubbard also holds the opinion as to causation just expressed herein, but does not know who originated it.) If this explanation be the true one, it follows that we should see the high, narrow arch in its extreme development mainly in the cases where tonsillar hypertrophy with adhesions to the pillars of the fauces is particularly well marked, and beginning while the bones are still young and soft enough to be capable of readily yielding to such force; and this I believe to be true, so far as my own observations go.

(*b*) The second factor in causing the narrow arch is so obviously important that no one can gainsay it. Once let this condition be begun by the cause just studied, and presently we will find that the narrow superior dental arcade will begin to articulate towards the inner or lingual side of the inferior dental arcade; for the lower jaw is not especially involved, and develops naturally, therefore becoming wider in the transverse measurement between its alveolar process than the upper jaw. As soon as such abnormal articulation has well begun, every time the teeth are firmly opposed they will tend to maintain the narrowness of the upper arch, and even to exaggerate it.

(*c*) The third factor in causation of this malformation of the upper jaw has to do directly with the pharyngeal obstruction and not now with the faucial. It seems an axiom in nature that any organ, any function, not put into use becomes atrophied and shrunken. Of this numerous instances will occur to you all. With the pharynx so filled up that the child has become a mouth-breather, the nasal air-passages have no longer the same *raison d'être.* They become comparatively useless in the economy, and from all sides nature begins to close in upon this waste space, largely perhaps from atmospheric pressure. The rising of the arch of the palate, which is also the floor of the nose, is a step in that process.

(*d*) In the case of young mouth-breathers the still soft bones of the upper jaw may well be expected to yield in some degree to the downward and continual traction of the soft parts of the sides of the face, pulled upon by the weight of the fallen lower jaw. The effect of this must necessarily be to bring the sides of the arch nearer together.

(*e*) In a recent most interesting study of the stigmata of degeneration, Dr. Frederic Peterson, one of our highest authorities, has discussed many of these, and among others he includes abnormally shaped palates, specifying the high, narrow, or Gothic roof of the mouth, the hip-roofed shape, the abnormally flattened, and a few others. I do not mean to have you infer that either he or I believe that more than a small minority of such palates represent such stigmata; but a few do, and hence we must include this factor among our causes. In some instances, though not indicative of degeneration, the narrow arch, or other unusual shape is unquestionably a matter of family inheritance, just as the shape of the nose is for example.

(*f*) Dr. J. B. Littig has pointed out that normally the tongue against the roof of the mouth supports the latter and maintains its normal shape while the bones are soft, in nose-breathers. But in mouth-breathers the tongue no longer can serve this useful function.

3. The third of the bad results of a stomatological nature, to which I alluded a few minutes ago, was dentition irregular in order and imperfect in quality. This it would seem can readily be explained since it is in the same category with the numerous other physiological activities adversely affected by insufficient oxidation, and by continually poisoned salivary and mucous secretions, with consequent anæmia and malnutrition.

4. Regarding a fourth group of symptoms caused by the disease we are studying, I would allude to the readiness wherewith the teeth of these children decay, and especially those teeth which are hindmost, thus lying nearest to the poison-filled tonsils or pharyngeal vegetations.

Quite recently, in talking informally of tonsillar troubles, a member here present volunteered the remark that he was sure he had observed this, and two other members confirmed the statement from their own experience. It will be interesting to ascertain the general views upon the point, in the discussion hereafter.

The diagnosis: How shall this be made? Of the six way-stations upon the ellipse of Waldeyer's lymphoid ring, the lowermost three are open to ocular inspection with some degree of ease,—*i.e.*, the tonsil of the tongue on its upper surface and quite close to the epiglottis, and the two true or faucial tonsils.

*A*, Luschka's tonsil; *B*, Eustachian or tubal tonsils; *C*, true or faucial tonsils; *D*, glossic or tonsil of the tongue.

The symptoms produced by hypertrophy of the glossic tonsil need not concern us in this paper. The other three way-stations are all pharyngeal ones,—namely, the uppermost or Luschka's tonsil, and the two tubal ones, or cushions of the Eustachian tubes.

Of course, the scientific and exact way of diagnosis is to examine the pharynx by aid of the forehead mirror, and the laryngoscopic or the post-nasal, which is a little mirror just such as you employ for help in your own work. But the educated finger-tip is quite sufficient though a more unpleasant way to determine absolutely the need for operation.

One can recognize with ease in an instant, after a little practice, the absence of the smooth, slippery, healthy mucous membrane, resembling closely in feel the inside of the cheek,—the buccal mucous membrane,—and instead, the presence of the mushroom or cauliflower growth varying greatly in consistency according to age, duration, and rapidity of development. Also the doctor can determine at the same moment whether one or both of the passages of the posterior nares be obstructed.

A very simple means of diagnosis, requiring but a second or two of time and no experience, is, that if the examiner's soft finger-tip, with nail trimmed close to the quick, produce a naso-pharyngeal hemorrhage, there is surely an abnormal and excessive degree of vascularity there, calling at least for a surgical opinion. There should no more be bleeding from the pharyngeal vault swept lightly by the finger-tip than from the tongue or the buccal mucous surfaces under like circumstances. To be sure, malignant growths and certain other diseases may bleed thus upon touch, but these are very rare by comparison, and of course also need a consultant's opinion.

But aside from any direct examination of the space behind the curtain of the soft palate, if you find a child who is a mouth-breather at most times, who snores when asleep, whose utterance tends to be thick and resembling in its faulty consonants the pronunciation caused by severe cold in the head, whose expressionless face and open mouth give him a stupid look, with even less than all these together, you can be practically sure of your diagnosis. (Of course, I assume that there is no obstruction of the nasal passages to be observed from the front.) With such a picture we commonly expect to find also enlarged faucial tonsils; and their presence accordingly adds to your certainty.

It is worth noting *en passant* that a baby who is very subject to coryza, and "snuffles" most of the time, is probably either syphilitic by inheritance or already afflicted with pharyngeal lymphoids.

In children old enough to understand and follow directions we are able to try the Valsalvian test, of closing the nostrils and trying to have them blow air through their Eustachian tubes, which any normal person can learn to do, feeling the air distend the ear-drums. Not to be able is often indicative of obstruction at the mouths of these tubes, and explains why growths here are known to be the most frequent cause of deafness; for the air pressure should in the healthy tympanum be equal upon both sides of the ear-drum.

Of course, this is a test as to the Eustachian tubes, and not merely as to the presence of vegetations in the pharynx which may be present and growing in such a way as not to press on the tubes.

Clifford Allbutt states that the very worst degrees of depressed ear-drums are found in bad cases of pharyngeal lymphoid growths, and that these children are the ones who, when stricken with diphtheria or scarlet fever, quite regularly develop suppurative otitis media and perforations.

### PREVENTION AND TREATMENT.

I know of no means whereby in a catarrhal climate, such as that of the northeast American seaboard, one can be assured of success in preventing lymphoid developments. Of course, local cleanliness is of the utmost importance. The hygiene of the nose should be taught as carefully as that of the mouth, and how the nose may with safety be cleansed; for if done improperly, as we all know, syringing is capable of causing damage to the ears, by forcing infected mucous discharges up the tubes.

One point in prevention may seem to you somewhat heterodox, and yet upon after-thought will, I believe, commend itself to you, —namely, that so far from endeavoring to break a baby of the habit of thumb-sucking, in our climate at least, it is rather to be encouraged; for it is obviously true that a thumb-sucking child cannot be mouth-breathing at the same time, and that consequently the habit promotes the natural function of the nose and nasopharynx in respiration, tending to keep these passages free. Also so far as a slight vacuum is produced in the mouth during the sucking, between the tongue and hard palate, this should tend to bring down the arch of the hard palate through atmospheric pressure from above,—that is, air within the nose. Of course, it is plain that there are certain disadvantages too; possible protrusion of the upper front teeth, for example; but I am alluding just now to naso-pharyngeal affairs, and upon the score of these am gallantly defending the little ones' chief comfort in life when aggrieved and unhappy.

TREATMENT.

This is solely operative. I will waste no time over discussing palliative measures. The operation of tonsillotomy is performed in a few seconds with the guillotine of various modifications. We also need to separate by another instrument the frequently adherent pillars. In very rare cases the shape of the tonsil is flat and diffuse, rendering amputation impossible and demanding the electric or actual cautery point, again and again, for its diminution and absorption.

As to anæsthesia, I prefer the local application of a solution of eucaine B rather than cocaine, for the reason that eucaine B does not shrink the growth, for it does not contract arterioles. Cocaine *does*, very distinctly, so that one cannot remove, after use of cocaine, so much as is desired, because of this retraction due to sudden anæmia. Of course, the latter—*i.e.*, the anæmia—is a good point as to checking bleeding. However, hemorrhage is rarely excessive, and gargling with very hot water usually suffices. Not to carry this paper to greater lengths, I do not discuss the treatment of such occasional instances of further bleeding. Upon this point the reader is referred to the author's article upon "Tonsillar

Hemorrhage: Its Prevention and Treatment," in the *Medical Record,* December 17, 1892; also *New York Medical News,* May 20, 1899. It is enough to say that the operation is entirely safe.

Regarding the ablation of pharyngeal lymphoids, simple as all agree that this is in skilled hands, opinions differ widely as to the best technique. I will quote Seifert (*Die ärtzliche Praxis,* 1898, xi. 81): "There is hardly another form of disease in which individual views regarding the method of operation, as well as anæsthetic to be employed, are so diverse as in adenoid vegetations." This prominent specialist prefers chloroform, only a very light seminarcosis being allowed, and the child held sitting upright and leaning forward, the blood running thus out of the mouth and nose. Certain others are cruel enough to use no anæsthesia at all. Cocaine or eucaine are nearly useless because of the flow of blood which promptly washes them out of the tissues. Indeed, I know no small operation in which there is, just for an instant, such a gush of hemorrhage as that at the moment of detaching these very vascular growths; but it ceases as promptly as it comes, and is not to be feared.

Personally I do not consider it safe to give chloroform in the upright position. My own choice is for chloroform in this operation, as in most, provided the anæsthetist be skilled; otherwise ether. It is generally admitted that in childhood chloroform is safer than otherwise; and with care it may even be given during sleep, thus preventing all excitement, the child sliding from natural into anæsthetic sleep. The child is gagged and then put in Rose's position, in which the head is allowed to drop down and backward beyond the end of the table; thus the blood cannot run towards the larynx, being directed by gravity out of the nose and mouth.

As Dr. Delavan, among others, has pointed out, there is more than a theoretical danger of inhaling a blood-clot and thus choking, if the position of the child renders this possible. It is very likely that the gentlemen here present to discuss this paper may each have his own choice herein; as also regarding the preferable instrument. Gottstein's curette, a kind of ring-knife, is very safe, and is popular for this purpose; though post-nasal forceps of various curves and shapes, and a few other tools, are occasionally employed, —Löwenberg's, or Hartmann's, or Trautmann's forceps to clear out Rosenmüller's fossa, for example.

In little babies the vegetations may sometimes be found so soft that even a strong and long finger-nail will suffice for their removal. With the flowing blood and necessity for speedy work,—for bleeding only ends with detachment of the growths,—it is hardly necessary to say that the operation is not done with mirrors and reflected light, but entirely by sense of touch; the left forefinger guiding the action of the curette or of the forceps.

The after-treatment is very simple. There is but slight discomfort, for the operated surfaces are above and behind the hanging palate, and deglutition does not bring the food in contact with any raw part. Any ordinary sore throat often causes much more annoyance than these little people feel the next day. They usually are kept recumbent only twenty-four hours, assuming that the circulation is normal. If any unpleasant odor whatever be noted after a day or two, I gently syringe the nose with normal salt solution, warm; otherwise I do not disturb the healing surfaces which nature is at work upon.

The child must be encouraged to use the natural breathing passages now. Quite as a habit, mouth-breathing may otherwise continue, to his detriment. But *before* this operation, it is simple cruelty to demand that a mouth-breathing child shall keep his lips closed. It would mean a partial suffocation.

*Does the disease recur?* Very rarely, when the operation is well performed; and the same is true of a re-hypertrophy of the amputated tonsil. The cases of recurrence are so excessively rare as to be a negligible quantity, not over one per cent. at most.

### IN CONCLUSION.

If we admit that the points I have made are well and truly taken,—that in sundry ways tonsillar growths, both oral and pharyngeal, are bad for the stomatological welfare of the dentists' little patients,—then it surely follows that dentists have a duty to perform in urging upon the parents of such patients the need of surgical intervention for both prevention and cure,—the removal of faucial tonsils and careful separation of all adhesions here, to avoid the narrowing of the palatine arch; the ablation of pharyngeal tonsils to prevent such an arch, and also ill-development of the whole upper jaw and faulty dentition; the removal of such growths because they prevent sufficient oxidation of the blood, also because they are culture-cabinets for microbes innumerable, continually supplying in their deep crypts or irregular interspaces poisonous ptomaines and toxines from life and death processes of these little enemies which shoot with poisoned arrows, thus vitiating the victim's blood. In both these last ways are induced anæmia and vital weakness, whereby dentition, in common with other important physiological processes, suffers and is performed but indifferently well at best, and at worst very badly.

These things being by you made clear to the parents, and that the necessary operation is in no sense a dangerous one, I feel sure that few, indeed, among the more intelligent of these will fail to accept thankfully the means thus advised, and in later years to acknowledge in consequence a fresh debt of gratitude to that excellent friend, the conscientious and cultured family dentist.

## Figures and Tables

**Figure f1:**